# Muscle quality index and cardiovascular disease among US population-findings from NHANES 2011–2014

**DOI:** 10.1186/s12889-023-17303-1

**Published:** 2023-12-01

**Authors:** Yanlin Chen, Weidong Lin, Lu Fu, Huiyi Liu, Shuyu Jin, Xingdong Ye, Sijia Pu, Yumei Xue

**Affiliations:** 1 Department of Guangdong Cardiovascular Institute, Guangdong Provincial People’s Hospital (Guangdong Academy of Medical Sciences), Southern Medical University, Guangzhou, 510080 China; 2 The Second School of Clinical Medicine, Southern Medical University, Guangzhou, 510515 China; 3 School of Medicine, South China University of Technology, Guangzhou, 510006 China

**Keywords:** Muscle quality index, Cardiovascular Disease, Resistance exercise, National Health and Nutrition Examination Survey

## Abstract

**Background and objective:**

Cardiovascular disease (CVD) is the leading cause of morbidity and mortality in the United States. However, current evidence on the association between muscle quality and CVD is limited. This study investigates the potential association between the muscle quality index (MQI) and the prevalence of CVD and CVD-related mortality.

**Methods:**

Participants were selected from the National Health and Nutrition Examination Survey (NHANES) 2011–2014. Data on mortality and causes of death were obtained from the National Death Index (NDI) records through December 31, 2019. Statistical analysis used in this study, including weighted multivariable linear and logistic regression, cox regression and Kaplan-Meier (K-M) analysis, to estimate the association between MQI and all-cause mortality as well as CVD mortality. In addition, subgroup analysis was used to estimate the association between MQI and CVD subtypes, such as heart attack, coronary heart disease, angina, congestive heart failure, and stroke.

**Results:**

A total of 5,053 participants were included in the final analysis. Weighted multivariable linear regression models revealed that a lower MQI.total level was independently associated with an increased risk of CVD development in model 3, with t value =-3.48, 95%CI: (-0.24, -0.06), *P* = 0.002. During 5,053 person-years of 6.92 years of follow-up, there were 29 deaths from CVD. Still, the association between MQI.total and CVD mortality, as well as all-cause mortality did not reach statistical significance in the fully adjusted model (HR = 0.58, 95% CI: 0.21–1.62, *P* = 0.30; HR = 0.91, 95% CI:0.65,1.28, *P* = 0.59, respectively). Subgroup analysis confirmed that MQI.total was negatively associated with congestive heart failure (OR = 0.35, 95% CI = 0.18,0.68, *P* = 0.01).

**Conclusion:**

This study highlights the potential of MQI as a measure of muscle quality, its negative correlation with congestive heart failure (CHF). However, MQI was not very useful for predicting the health outcomes such as CVD and mortality. Therefore, more attention should be paid to the early recognition of muscle weakness progression in CHF. Further studies are needed to explore more effective indicator to evaluate the association between muscle quality and health outcomes.

**Supplementary Information:**

The online version contains supplementary material available at 10.1186/s12889-023-17303-1.

## Introduction

Cardiovascular disease (CVD) is the leading cause of morbidity and mortality in the United States [1]. In 2017, CVD accounted for approximately 859,125 deaths in the United States [2]. Additionally, the total cost of CVD in the United States was about $351.2 billion between 2014–2015 [2]. Although traditional risk factors, such as hypertension, obesity, hyperglycemia and smoking, have been well-qualified, other factors that are associated with CVD have been gradually recognized, including muscle mass, strength and quality [3].

Skeletal muscle consists of 40–50% of total body weight [4]. With increasing age, muscle mass declines by 0.37% and 0.47% per year in women and men, respectively [5]. Interestingly, the loss of muscle mass and strength does not mean a decrease in body weight, as the lost muscle may be replaced by nonfunctional fat, such as visceral fat, leading to muscle weakness [6]. Body muscle is metabolically active, which is identified as anti-hyperglycemia and anti-inflammation [7]. Recent clinical studies reported that the reduced muscle mass could be much higher in CVD patients compared with healthy population [8, 9]. Hajahmadi et al. found that 47.3% of patients with dilated cardiomyopathy, with an average age of 37 years, exhibited muscle wasting [10]. Similarly, patients who received transcatheter aortic valve implantation (TAVI) were followed for 12 months, and it was found that those with the lowest psoas muscle mass showed the highest new-onset atrial fibrillation and mortality [11]. Therefore, the muscle condition may serve as a novel predictor for prognosticating and evaluating the risk of CVD. Muscle mass can be measured by magnetic resonance imaging or dual-energy X-ray absorptiometry (DXA) scans. Handgrip strength (HGS) is a direct measure of arm strength and an indirect evaluation of overall muscular strength [3] (HGS can be well correlated with the whole muscle strength [12]). However, muscle wasting does not synchronize with the loss of muscle strength, as the loss of muscle strength is faster than muscle wasting [13]. The muscle quality index (MQI) is used to describe the muscle strength per unit of muscle mass and has been recognized as an indicator to access the body muscle [14, 15]. Several methodologic approaches have been employed to calculate MQI values, which have been discussed in previous articles [16–19].

Since both muscle mass and muscle strength have been shown to be relative to disability risk and CVD mortality, large and representative data are needed to identify these correlations. We acknowledge there are various methods to evaluate body muscle quality; thus, in this study, we adopted Lopes, L. et al. to introduce the MQI method to explore the relationship between MQI and health outcomes (CVD and mortality) [20]. We hypothesized that MQI plays a role in cardiovascular health.

## Methods

### Data collection

Data used in this cross-sectional and longitudinal study was from the National Health and Nutrition Examination Survey (NHANES). NHANES is a large-scale, ongoing and representative healthy survey of the U.S. civilians aged over 2 months [21]. NHANES has been approved by the National Center for Health Statistics (NCHS) Ethics Review Broad (ERB) and participants have written consent. The detailed protocol of data collection is described at http://www.cdc.gov/nchs/nhanes.

**Fig. 1:**
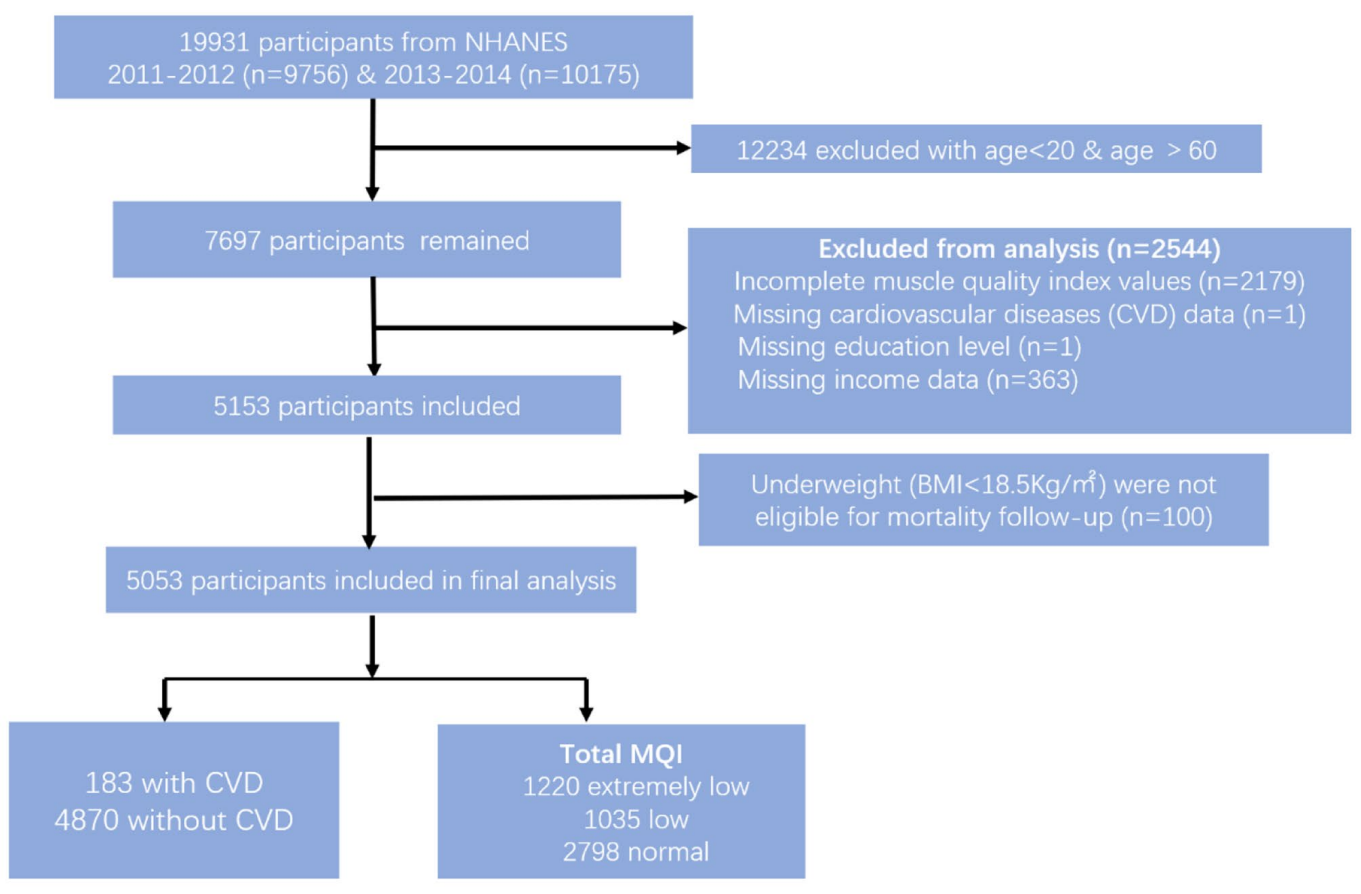
The flowchart of study sample selection form NHANES 2011–2012 and 2013–2014

Due to the MQI test being only available in the 2011–2014 period, thus, the dataset was collected from these 2 cycles of NHANES, 2011–2012 and 2013–2014. And we excluded participants below 20 and over 60, as the body composition data was unavailable. In addition, the study excluded the participants who lacked CVD data and incomplete covariates (Fig. 1).

### Exposure measurement methods

According to Lopes, L. et al. introduced the method to calculate MQI [22]. MQI_Arm_ (kg/kg) was the ratio between dominant HGS (measured by a Takei dynamometer) and dominant arm appendicular skeletal muscle mass (arm ASM accessed by DXA) [22]. In the same way, MQI_App_ (kg/kg) was the ratio of dominant HGS/ASM (ASM has been defined by the sum of four limbs of lean soft tissue); and the MQI_Total_ was the ratio of HGS sum/ASM (HGS sum was the sum of the dominant hand and the non-dominant hand) [22]. The continuous variable of MQI_Total_ has been transferred into categorical variable based on what Lopes, L. et al. reported; the MQI_Total_ cut-off values were established for both different ethnicities and sexes, which were normal, low and extremely low MQI_Total_ groups [22]. The rationale for use of the MQI cutoffs values was: previous studies showed variations in HGS and body composition across different sexes and racial [22–24], suggesting a sex- and population-specific MQI cut-off values should be established. Therefore, low MQI_Total_ were defined as 1 standard deviation (SD) bellow the mean of young adult reference, and extremely low MQI_Total_ defined as 2 SD below the young adult reference mean (the young adult group including male and female, age from 20 to 39 years old, and with normal BMI range), more details were shown in Supplementary Table 1.

### Outcome variable selections

The outcomes selection in this study were CVDs and mortality. Reported or self-admitted physician diagnoses obtained CVD. Self-admitted CVD was assessed by asking the following questions: “Has a doctor or other health professional ever told you that you have a heart attack/ coronary heart disease/ angina / congestive heart failure/ stroke?” The answer could be “Yes/No/didn’t know”, and “Yes” was considered as a CVD patient; the “didn’t know” participants would be excluded. Mortality due to all-cause and CVD was determined by connecting to the National Death Index (NDI) until December 31, 2019. The ICD-10 (codes I00-I09, I11, I13, I20-I51, or I60–I69) was employed to identify CVD mortality. The “2019 Public-Use Linked Mortality Files” were available on the NCHS website (https://www.cdc.gov/nchs/data-linkage/mortality-public.htm). We started from MQI-related data collection and ended on or before December 31, 2019. In addition, participants who did not have a matching death record were considered to be alive throughout the entire follow-up period.

### Covariates extraction

Covariates that could potentially affect the relationship between MQI and the risk of CVD/ CVD mortality were collected through interviews and medical examinations. The covariates have been divided into continuous variables and categorical variables. Continuous variable was the age; categorical variables including ethnicity (Non-Hispanic White, Non-Hispanic Black, Mexican American and other), sex (female, male), hypertension (no/yes), diabetes (no/yes), smoking status (no/yes), alcohol use (no/yes), educational level (less than 9th grade, 9 − 11th grade, high school grad/GED or equivalent, some college or AA degree, college graduate or above), body mass index < 18.5 was excluded in the analysis due to underweight (BMI, 18.6–25, 25–30, ≥ 30), family poverty-income ratio (PIR, low income < 1, 1 ≤ middle income < 3, high income ≥ 3).

Evidence showed that the moderate to vigorous aerobic physical activity (MVPA) and sedentary behavior were associated with CVD and mortality [25, 26], which as potential confounders in this study. Participants completed the Global Physical Activity Questionnaire (GPAQ), developed by the World Health Organization (WHO) [27], gathers information on sedentary behavior and the amount of time individuals spend per week engaging in physical activity across three domains: leisure, work, and travel. It assesses both moderate activities like brisk walking or cycling and vigorous activities like running or football. This self-report questionnaire consists of 16 questions. According to the Physical Activity Guidelines from US 2018 [28], MVPA was classified into categorical variables: low (< 150 min/week), moderate (150–300 min/week), and high (> 300 min/week) [29]. In addition, daily sitting time was extracted by asking participants, “On a typical day, how much time usually spend sitting at school, at home, getting to and from places, or with friends including time spent sitting at a desk, traveling in a car or bus, reading, playing cards, watching television, or using a computer, but did not include time spent sleeping?” Based on previous studies [30, 31], the daily sitting time was converted to hours/day (h/d), and divided into 4 groups (0 to < 4, 4 to < 6, 6 to 8, and ≥ 8).

### Statistical analysis

Statistical analyses were performed with R software (version 4.3.0). Proper weighting procedures were applied with the guidance of NHANES guidelines to derive accurate estimates that could represent the U.S. population, as the complex survey design. The weights calculation for 2011–2014 was created by taking one-half for each participant who was sampled in 2011–2012 and one-half for each participant who was sampled in 2013–2014 [1/2 * WTMEC2YR_(2011−2012)_ + 1/2 * WTMEC2YR_(2013−2014)_, WTMEC2YR is weight variable for 2011–2014]. Continuous variables were presented as means and standard error (SE), and categorical variables were presented as frequency plus percentage. To compare different variables, t-tests and Mann-Whitney U tests were employed for continuous variables, the chi-square test was used for categorical variables. Weighted linear regression analysis was performed to explore the association between MQI_Total_ and CVD. Meanwhile, weighted logistic regression was conducted to further validate the relationship between MQI_Total_ and CVD, estimating odds ratios (OR) with corresponding 95% confidence intervals (CI). Four analyzes models have been established to remove covariates affect, model 1 without covariate adjustment; Model 2 adjusted with physical activity; Model 3 adjusted with sex, age, ethnicity, and physical activity; Model 4 was further adjusted with hypertension, diabetes, smoking status, alcohol use, educational level, poverty-income ratio (PIR), and body mass index (BMI), and daily sitting time. All analyzed data were used for the cross-sectional analyses in the present study. Furthermore, participants were followed-up, and the association between MQI_Total_ and mortality was evaluated using cox regression and Kaplan-Meier (K-M) analysis with time-to-events as the time variable, estimating the hazard ratios (HR) and 95% CI. Subgroup analyses were performed to estimate the association between MQI.total and CVD subtypes (angina, stroke, heart attack, coronary heart disease, congestive heart failure). All statistical tests were two-sided, and a significance level of *P* < 0.05 was considered statistically significant.

## Results

This cross-sectional and longitudinal study enrolled 19,931 participants in the NHANES from 2011 to 2014. Among them, 12,234 participants were excluded with age < 20 and age > 60 as the body composition data was not available. In addition, 2,179 were excluded due to incomplete MQI values. After missing covariates were eliminated from the study, 5,053 participants were enrolled in the final analysis (shown in Fig. 1). The 5,053 NHANES participants with valid MQI values represented approximately 11.93 million noninstitutionalized residents in the USA. Population characteristics of the study are shown in Table 1. Among the participants, 183 had CVD and 4,870 did not. The prevalence of CVD in females and males was 2.83%(N = 86) and 3.45%(N = 97), respectively. The average age of non-CVD participants was 39.12 ± 0.41, and CVD was 48.47 ± 0.78. Compared to participants without CVD, those with CVD tended to be older, have lower education levels, engage in unhealthy habits (smoking and drinking), and have a higher prevalence of risk factors (hypertension, diabetes, and obesity). Most importantly, participants diagnosed with CVD had lower muscle mass and muscle strength, as indicated by lower MQI values (MQIc.arm, MQIc.app and MQIc.total), compared to the population without CVD.

**Table 1:** The general characteristics of the population in NHANESE 2011–2014, statistical analysis was presented with Mean ± SE and Percentage (frequency). CVD, cardiovascular disease. BMI, body mass index. MQIc.arm, categorical value of muscle quality index for arms. MQIc.app, categorical value of muscle quality index for four limbs. MQIc.total, categorical value of whole-body muscle quality index

Variable	Total (5053)	Non-CVD (4870)	CVD (183)	**P** value
**Age**	39.41 ± 0.40	39.12 ± 0.41	48.47 ± 0.78	**< 0.0001**
**Sex**				0.25
Female	2464(48.76)	2378(97.17)	86( 2.83)	
Male	2589(51.24)	2492(96.55)	97( 3.45)	
**Ethnicity**				0.31
Non-Hispanic White	2034(40.25)	1955(96.87)	79( 3.13)	
Non-Hispanic Black	1097(21.71)	1047(95.86)	50( 4.14)	
Mexican American	612(12.11)	590(97.12)	22( 2.88)	
Other	1310(25.93)	1278(97.32)	32( 2.68)	
**Education Level**				**0.002**
Less than 9th grade	229(4.53)	219(96.60)	10( 3.40)	
9-11th grade	610(12.07)	571(94.19)	39( 5.81)	
High school graduate/GED or equivalent	1074(21.25)	1025(95.52)	49( 4.48)	
Some college or AA degree	1703(33.7)	1646(96.85)	57( 3.15)	
College graduate or above	1437(28.44)	1409(98.55)	28( 1.45)	
**Smoke**	1204(23.84)	1128(93.92)	76( 6.08)	**< 0.0001**
**Poverty Level**				**< 0.001**
low income	1736(34.36)	1653(95.22)	83( 4.78)	
middle income	1647(32.59)	1580(96.16)	67( 3.84)	
high income	1670(33.05)	1637(98.34)	33( 1.66)	
**Alcohol User**	3687(76.94)	3573(97.39)	114( 2.61)	**0.01**
**Hypertension**	1431(28.32)	1306(92.54)	125( 7.46)	**< 0.0001**
**Diabetes**	680(13.46)	618(90.98)	62( 9.02)	**< 0.0001**
**BMI (kg/m2)**				**0.001**
18.5–25	1617(32)	1581(98.35)	36( 1.65)	
25–30	1625(32.16)	1570(97.01)	55( 2.99)	
>=30	1811(35.84)	1719(95.37)	92( 4.63)	
**MQIc.arm**				**< 0.0001**
extremely low	734(14.53)	680(93.91)	54( 6.09)	
low	1059(20.96)	1010(96.21)	49( 3.79)	
normal	3260(64.52)	3180(97.87)	80( 2.13)	
**MQIc.app**				**< 0.0001**
extremely low	751(14.86)	692(93.57)	59( 6.43)	
low	1118(22.13)	1078(97.02)	40( 2.98)	
normal	3184(63.01)	3100(97.72)	84( 2.28)	
**MQIc.total**				**< 0.0001**
extremely low	1220(24.14)	1139(94.60)	81( 5.40)	
low	1035(20.48)	994(96.65)	41( 3.35)	
normal	2798(55.37)	2737(97.99)	61( 2.01)	
**Daily sitting time (hour)**				0.07
<4	1058(20.94)	1017(96.65)	41( 3.35)	
4 to < 6	1119(22.15)	1090(97.85)	29( 2.15)	
6 to 8	1591(31.49)	1517(95.83)	74( 4.17)	
>8	1285(25.43)	1246(97.31)	39( 2.69)	
**Physical Activity**				0.06
low	671(16.26)	649(97.87)	22( 2.13)	
moderate	682(16.53)	661(96.28)	21( 3.72)	
high	2773(67.21)	2691(97.73)	82( 2.27)	

As shown in Table 2, we investigated the association between MQI.total values and the outcome of CVD by using linear regression. In Model 1 (no covariate was adjusted), relative to non-CVD participants, CVD participants had significantly lower MQI.total values compared to non-CVD participants (mean difference, 0.28 kg/kg; *P* < 0.0001); in Model 2 (physical activity were adjusted), CVD participants had lower MQI.total values compared with non-CVD (mean difference, 0.21 kg/kg; *P* < 0.0001); in Model 3 (sex, age, ethnicity, and physical activity were adjusted), CVD participants still exhibited lower MQI.total values (mean difference, 0.15 kg/kg; *P* = 0.002); After further adjustment in Model 4 (sex, age, ethnicity, physical activity, hypertension, diabetes, smoking status, alcohol use, educational level, PIR, BMI, and daily sitting time were further adjusted), MQI.total values was not independently associated with CVD (mean difference, 0.09 kg/kg; *P* = 0.09). Logistic regression (Table 3) was performed to validate the MQI.total level was negatively associated with the odds ratio (OR) of CVD [Model 1: OR (95% CI) = 0.47 (0.35–0.63), *P* < 0.0001; Model 2: OR (95% CI) = 0.55 (0.42–0.72), *P* < 0.001; Model 3: OR (95% CI) = 0.65 (0.50–0.84), *P* = 0.002]. However, after fully adjustment in Model 4, the MQI.total values was not independently associated with CVD [Model 4: OR (95% CI) = 0.79 (0.49–1.29), *P* = 0.31].

**Table 2:** Linear regression results of associations between CVD and MQI.total. Model 1 was analyzed without covariate adjusted; Model 2 adjusted with physical activity, Model 3 adjusted with sex, age, ethnicity, and physical activity; Model 4 was further adjusted with hypertension, diabetes, smoking status, alcohol use, educational level, poverty-income ratio (PIR), and body mass index (BMI), and daily sitting time. CVD, cardiovascular disease. BMI, body mass index. MQI.total, whole-body muscle quality index. CI, confidence interval. PIR, poverty-income ratio

	Model 1 Estimate (95% CI)	*P* value	Model 2 Estimate (95% CI)	*P* value	Model 3 Estimate (95% CI)	*P* value	Model 4 Estimate (95% CI)	*P* value
CVD								
No	Ref		Ref		Ref		Ref	
**Yes**	-0.28 (-0.40, -0.17)	**< 0.0001**	-0.21(-0.31, -0.12)	**< 0.0001**	-0.15 (-0.24, -0.06)	**0.002**	-0.09 (-0.20, -0.02)	0.09

**Table 3:** Association of MQI.total with the prevalence of CVD, all-cause mortality, and CVD-related mortality among US population, NHANES, 2011–2014. Model 1 was analyzed without covariate adjusted; Model 2 adjusted with physical activity, Model 3 adjusted with sex, age, ethnicity, and physical activity; Model 4 was further adjusted with hypertension, diabetes, smoking status, alcohol use, educational level, PIR, and BMI, and daily sitting time. CVD, cardiovascular disease. CVD, cardiovascular disease. MQI.total, whole-body muscle quality index. BMI, body mass index. PIR, poverty-income ratio. CI, confidence interval

	Model 1 OR/HR(95%CI)	*P*-value	Model 2 OR/HR(95%CI)	*P*-value	Model 3 OR/HR(95%CI)	*P*-value	Model 4 OR/HR(95%CI)	*P*-value
CVD								
No	Ref		Ref		Ref		Ref	
Yes	0.47(0.35,0.63)	**< 0.0001**	0.55(0.42,0.72)	**< 0.001**	0.65(0.50,0.84)	**0.002**	0.79(0.49,1.29)	0.31
**All-cause mortality**								
Alive	Ref		Ref		Ref		Ref	
Death	0.59(0.43,0.81)	**< 0.001**	0.68(0.51,0.90)	**0.01**	0.74(0.55,0.99)	**0.04**	0.91(0.65,1.28)	0.59
**CVD mortality**								
No	Ref		Ref		Ref		Ref	
Yes	0.33(0.17,0.65)	**0.001**	0.44(0.26,0.74)	**0.002**	0.51(0.31,0.82)	**0.01**	0.58(0.21,1.62)	0.3

During 5,053 person-years of follow-up (median follow-up, 6.96 years) from the NHANES 2011–2014, a total of 127 deaths were documented, with 29 deaths attributed to CVD. First, the multivariable adjustment was applied to analyze the association between MQI.total and all-cause mortality. The results revealed a negative association between MQI.total and all-cause mortality [Model 1: HR (95% CI) = 0.59(0.43,0.81), *P* < 0.001; Model 2: HR (95% CI) = 0.68(0.51,0.90), *P* = 0.01; Model 3: HR (95% CI) = 0.74(0.55,0.99), *P* = 0.04], although statistical significance was not achieved in the fully adjusted model 4 (shown in Table 3). In addition, the risk of CVD mortality increased with the decreased MQI.total values, shown in Table 3 [Model 1: HR (95% CI) = 0.33(0.17,0.65), *P* = 0.001; Model 2: HR (95% CI) = 0.44(0.26,0.74), *P* = 0.002; Model 3: HR (95% CI) = 0.51(0.31,0.82), *P* = 0.01]. However, after fully adjustment, the MQI.total values was not independently associated with CVD mortality [Model 4: HR (95% CI) = 0.58(0.21,1.62), *P* = 0.3]. In K-M analysis, participants with weaker muscle quality, especially those with extremely low MQI levels, exhibited the highest risk of all-cause and CVD mortality, although statistical significance was not achieved (shown in Supplementary Fig. 1).

Subgroup analyses of the association between MQI.total and CVD subtypes were shown in Table 4. After adjusting for physical activity (Model 2, Table 4), a negative association was found between MQI.total and individual CVDs, including angina and stroke [OR (95% CI) = 0.64(0.41,0.99), *P* = 0.04; OR (95% CI) = 0.50(0.31,0.83), *P* = 0.01, respectively). Importantly, MQI.total was significantly associated with congestive heart disease after adjusting for all covariates in model 4, [OR (95% CI) = 0.35(0.18,0.68), *P* = 0.01].

**Table 4:** Logistic regression was performed to study the associations of MQI.total with individual CVDs (Heart attack, Coronary heart disease, Angina, Congestive heart failure, Stroke) risk in US adults 2011–2014. Different models were adjusted by different covariables. CVD, cardiovascular disease. MQI.total, whole-body muscle quality index. CI, confidence interval

Individual CVD	Odds ratio (95% CI)	**P**-value
**Heart attack**		
Model 1	0.49(0.27,0.87)	**0.02**
Model 2	0.60(0.30,1.18)	0.13
Model 3	0.70(0.34,1.44)	0.31
Model 4	0.91(0.30,2.79)	0.86
**Coronary heart disease**		
Model 1	0.40(0.24,0.67)	**< 0.001**
Model 2	0.63(0.33,1.21)	0.16
Model 3	0.77(0.38,1.55)	0.44
Model 4	0.99(0.27,3.64)	0.99
**Angina**		
Model 1	0.48(0.25,0.92)	**0.03**
Model 2	0.64(0.41,0.99)	**0.04**
Model 3	0.76(0.49,1.18)	0.21
Model 4	1.06(0.45,2.49)	0.89
**Congestive heart failure**		
Model 1	0.24(0.13,0.44)	**< 0.0001**
Model 2	0.30(0.13,0.69)	**0.01**
Model 3	0.38(0.16,0.90)	**0.03**
Model 4	0.35(0.18,0.68)	**0.01**
**Stroke**		
Model 1	0.45(0.28,0.72)	**0.002**
Model 2	0.50(0.31,0.83)	**0.01**
Model 3	0.67(0.40,1.10)	0.11
Model 4	0.56(0.22,1.42)	0.19

## Discussion

To our knowledge, this study represents the first to use the MQI to investigate the relationship between muscle quality and the prevalence and mortality of CVD. According to the results of weighted linear and logistic multivariable regression analysis, although a weak negative correlation was observed between MQI.total and the prevalence of CVD, the fully adjusted model showed that low MQI.total did not predict the risk of CVD. In a study by Wu et al., sarcopenia, defined by low calf circumference, muscle strength, mass, and gait speed, was found to be associated with a higher risk of all-cause mortality [32]. Kitamura A et al. reported that Japanese older women and men meeting Asia criteria of sarcopenia had an increased risk of all-cause mortality [33]. A study enrolled 469,830 UK Biobank participants, sarcopenia, defined as slow gait speed and low muscle mass, was found to be associated with adverse outcomes, including the incidence and mortality of CVD, as well as all-cause mortality [34]. However, in our study, no association was observed between MQI.total and mortality after fully adjusting for potential confounders. Meanwhile, K-M analysis indicated that with the follow-up duration increased, individuals with poor muscle quality had a lower survival rate, although this did not reach statistical significance. Based on the current results, MQI.total was not a useful predictor for assessing the incidence of CVD and mortality. These results may be attributed to MQI may have some limitations in assessing muscle quality, as it relies on a ratio of handgrip strength and muscle mass. In some cases, individuals with both lower handgrip strength and lower muscle mass may paradoxically exhibit a higher MQI value, making it challenging to accurately evaluate the relationship between muscle quality and health outcomes (CVD and mortality). In addition, older adults are prone to CVD and mortality due to the biologic underpinning of aging [35]. In previous studies, the majority of participants with poor muscle quality were older; however, our study exclusively enrolled middle-aged participants, potentially introducing selection bias.

Subgroup analysis was further conducted, revealing that participants with CHF were more likely to have low muscle quality than the normal participants, while there was no association between the MQI.total and other individual CVDs in the fully adjusted model. Consequently, the MQI.total indicator was more sensitive in predicting the prevalence of CHF. Despite many different clinical tests and techniques, there was still no broadly available standard for the diagnosis of muscle wasting. In this study, MQI was introduced as a novel indicator of muscle quality in an attempt to explore the relationship between muscle wasting and health outcomes. However, the fully adjusted models showed that low MQI.total did not predict any health outcomes except for CHF. CHF is a systemic disease affecting approximately 2% of the population in the world [36, 37]. Muscle wasting has been considered as a frailty marker and has been linked to reduce survival in CHF patients [38]. Muscle wasting could induce ventilatory inefficiency and exercise intolerance in CHF patients [39]. The Studies Investigating Co-morbidities Aggravating Heart Failure (SICA-HF) enrolled 200 chronic HF patients and found that sarcopenia in HF patients with reduced ejection fraction was 20% higher than in healthy individuals [40]. Similarly, sarcopenia was also prevalent in HF patients with a preserved ejection fraction [41]. Therefore, it appears that muscle quality and CHF seem to be intertwined, affecting the progression and outcome of each other [42].

The significance of muscle quality in relation to cardiovascular health and overall well-being has garnered increasing recognition. Different from the well-known role of obesity in CVD and mortality (all-cause and CVD-related mortality), the impact of muscle quality on CVD risk and mortality is emerging and recognized nowadays. The underlying pathophysiologic mechanism between muscle quality and CVD risk and mortality is complex and needs further study. Based on the current study, chronic systemic inflammation plays a critical role in muscle wasting and deteriorating muscle quality, contributing to CVD and mortality [35]. Proinflammation cytokines such as interleukin(IL-6) and tumor necrosis factor-α (TNF-α) initiated muscle mitochondrial dysfunction, large amount of reactive oxygen species (ROS) production, leading to muscle proteolysis [35]. Meanwhile, risk factors of inflammation and oxidative stress for muscle health are also shared with CVD, leading to myostatin released from both myocytes and cardiomyocytes, establishing a vicious circle [43]. These findings provide evidence that ROS overload in muscles could initiate an inflammatory response that could affect the cardiovascular system.

In this study, we found that MQI for the whole body was negatively associated with CHF; thus, enhanced MQI level might reverse outcome. Physical exercise and nutrition intervention are two main strategies to improve MQI [35]. In Tang et al. study, they found that soy or whey protein consumption combined with moderate resistance exercise could be better than adopting either one individually to enhance muscle quality [44]. Additionally, oral testosterone supplementation has been shown to increase muscle mass and strength [45, 46], although its effect on CHF requires further investigation. Angiotensin-converting enzyme inhibitors (ACEIs), commonly used in CHF treatment, interestingly, not only improve cardiac function but also suppress inflammatory responses and improve mitochondrial function, thereby reducing muscle catabolism [47]. Consequently, ACEI is one of the cornerstones for CHF treatment and it could improve muscle quality [35].

This exploratory analysis provided some insight into potential risk factors (low muscle mass, strength and quality) for CVD and mortality (all-cause and CVD-related mortality), especially for CHF. The major strength of this study is the large, representative sample size of the US population allowed us to fully understand the relationship between MQI and CVD, as well as mortality. In addition, the study has adjusted a wide range of potential covariables and used different statistical methods to minimize bias and validate the results. We employed MQI.total for a quantitative assessment of muscle quality, allowing for a more precise evaluation of the relationship between muscle quality and CHF. Consequently, subgroup analysis suggested that MQI was specifically associated with CHF, offering potential management strategies for these patients. However, we acknowledge that there are several limitations to this study. First, study showed that MQI was not very useful for predicting the health outcomes such as CVD and mortality. previous studies reported that the prevalence of sarcopenia, CVD and CVD mortality was high in the elderly group, while this study only included 20-59-year-old participants due to incomplete exam information. Second, the outcome collection was based on self-reported, which might result in reporting bias. Further explore the association between MQI.app, MQI.arm and CVD was not performed in this study. Finally, the subgroup analysis couldn’t investigate the correlation between MQI and arrhythmias due to the unavailability of this outcome in the NHANES database.

## Conclusions

In conclusion, while MQI was not very useful for predicting the health outcomes such as CVD and mortality, a low MQI value has the potential to serve as a risk indicator for patients with CHF. Therefore, more attention should be paid to the early recognition of muscle weakness progression in CHF. Muscle weakness contributes to poor outcomes, thereby garnering public attention and motivating individuals to engage in appropriate resistance exercise, ultimately reducing the incidence of CVD and the mortality rate. However, further studies are needed to explore more effective indicator to evaluate the association between muscle quality and health outcomes.

### Electronic supplementary material

Below is the link to the electronic supplementary material.

Supplementary Material 1

## Data Availability

The datasets used in this manuscript are publicly available from the NHANES website: https://www.cdc.gov/nchs/nhanes/index.htm.

## Abbreviations

NHANES: National Health and Nutritional Examination Survey
NDI: National Death Index
NCHS: National Center for Health Statistics
MQI: Muscle quality index
CVD: Cardiovascular disease
MQIc.arm: Categorical value of muscle quality index for arms
MQIc.app: Categorical value of muscle quality index for four limbs
MQIc.total: Categorical value of whole-body muscle quality index
PIR: Poverty income ratio
MQI.total: Whole-body muscle quality index
MVPA: Moderate to vigorous aerobic physical activity
GPAQ: Global Physical Activity Questionnaire
