# Supplementary material for: Muscle quality index and cardiovascular disease among US population-findings from NHANES 2011–2014
Source: BMC Public Health. 2023 Dec 1;23:2388. doi: 10.1186/s12889-023-17303-1 (PMC10691039; doi:10.1186/s12889-023-17303-1)
Supplement: Supplementary file 1 — Supplementary Material 1 [file 12889_2023_17303_MOESM1_ESM.docx]

Supplementary Table 1 Cut-off values for normal, low and extremely low MQI_Total_ among study population based on young population.

|  | **MQI_Total_** | | |
| --- | --- | --- | --- |
| **Male** | **Normal** | **Low** | **Extremely Low** |
| Non-Hispanic White | >3.3 | 3.1-3.3 | ≤3.1 |
| Non-Hispanic Black | >3.5 | 3.1-3.5 | ≤3.1 |
| Mexican American | >3.6 | 3.1-3.6 | ≤3.1 |
| Others | >3.2 | 2.6-3.2 | ≤2.6 |
| **Female** |  |  |  |
| Non-Hispanic White | >3.4 | 3.1-3.4 | ≤3.1 |
| Non-Hispanic Black | >2.9 | 2.3-2.9 | ≤2.3 |
| Mexican American | >3.4 | 3.0-3.4 | ≤3.0 |
| Others | >3.2 | 2.8-3.2 | ≤2.8 |

Supplementary Figure 1. Kaplan-Meier (K-M) survival curves according to MQI.total for all-cause mortality (1-1) and CVD mortality (1-2). The unit of “Time” was month. MQI.total, whole-body muscle quality index. CVD, cardiovascular disease.


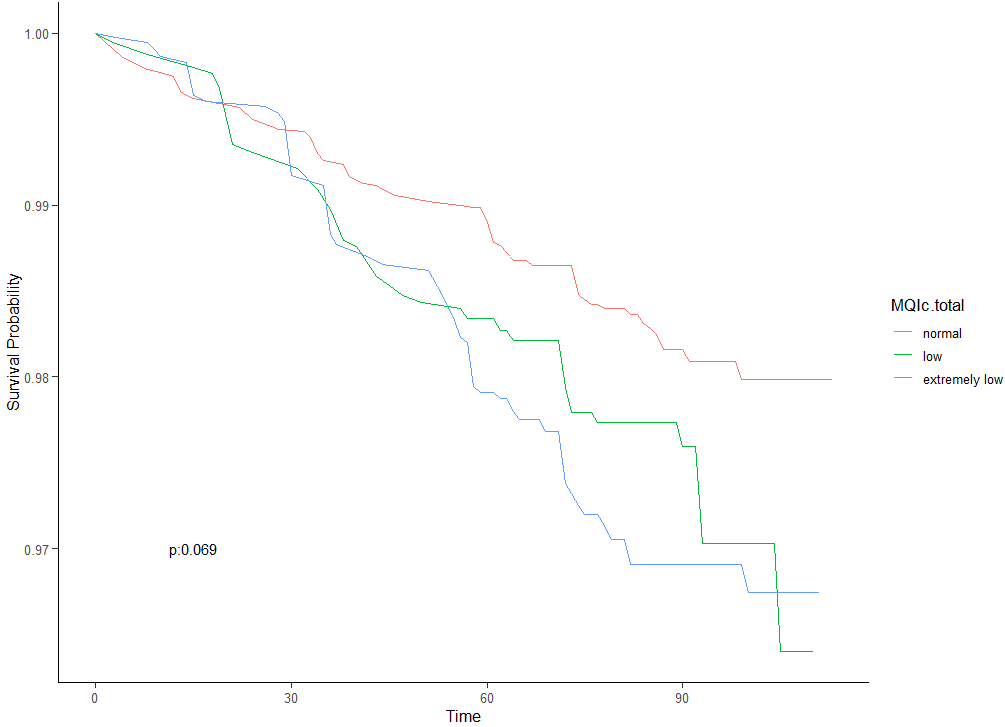


1-1


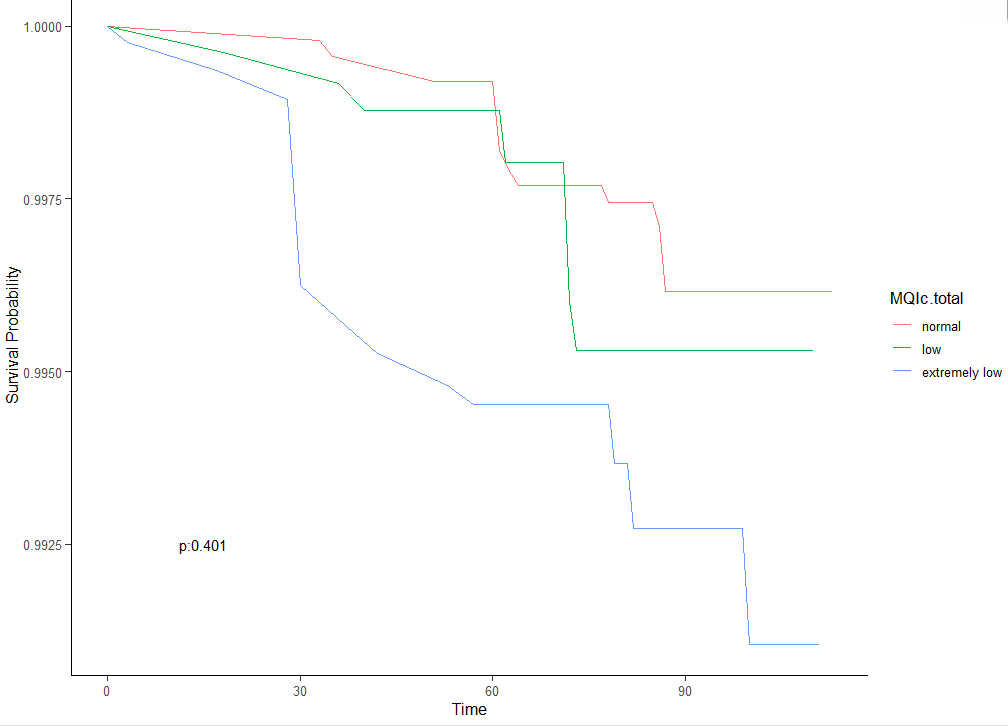


1-2
